# Estrogen-ERα signaling and DNA hypomethylation co-regulate expression of stem cell protein PIWIL1 in ERα-positive endometrial cancer cells

**DOI:** 10.1186/s12964-020-00563-4

**Published:** 2020-06-05

**Authors:** Zheng Chen, Hua-Jing Yang, Qin Lin, Min-Jiao Zhu, Ying-Ying Yu, Xiao-Ying He, Xiao-Ping Wan

**Affiliations:** 1 Department of Obstetrics and Gynecology, International Peace Maternity and Child Health Hospital, School of Medicine, Shanghai Jiao Tong University, No.910, Hengshan Road, Shanghai, 200030 China; 2 Shanghai Municipal Key Clinical Specialty, Shanghai, China; 3 Shanghai Key Laboratory of Embryo Original Diseases, Shanghai, China; 4 Department of Obstetrics and Gynecology, Shanghai First Maternity and Infant Hospital, Tong Ji University School of Medicine, No. 536, Changle Road, Shanghai, 200080 China

**Keywords:** Endometrial carcinoma, PIWIL1, ERα, Cell Proliferation, DNA methylation

## Abstract

**Background:**

We previously identified *PIWIL1* as an oncogene involved in endometrial carcinogenesis. However, the mechanism of Piwil1 mediated regulation of tumorigenesis remains poorly understood.

**Methods:**

The expression levels of target genes in endometrial cancer cells were detected by quantitative reverse transcription-PCR (RT-qPCR) and western blotting. Up- or down-regulation of ERα or PIWIL1 was achieved by transient transfection with expressing plasmids or short hairpin RNA (shRNA). Dual-luciferase reporter assays and chromatin immunoprecipitation (ChIP) were used to demonstrate the ERα bound to the half estrogen response element (half-ERE) located in *PIWIL1* promoter. The expression of PIWIL1 and ERα in endometrial carcinoma tissues were investigated using immunohistochemistry and RT-qPCR. The proliferation ability of cancer cells were evaluated by MTT. Methylation status of the *PIWIL1* promoter was detected by bisulfite sequencing PCR (BSP).

**Results:**

In the present study, we found that PIWIL1 mediated E_2_-stimulated cancer cell proliferation. In ERα-positive endometrial cancer cells, we demonstrated that estrogen-ERα signaling significantly up-regulated the expression of PIWIL1, which was mediated by binding of the ERα onto the *PIWIL1* promoter. Furthermore, we found that a half-ERE in the *PIWIL1* promoter was essential for ERα binding. The *PIWIL1* promoter was hypomethylated in ERα-positive endometrial cancer cells. Treatment with 5-aza-deoxycytidine (5-aza-dC) could up-regulate PIWIL1 expression.

**Conclusions:**

These findings uncover a novel molecular mechanism by which estrogen-ERα signaling and DNA hypomethylation co-regulate PIWIL1 expression. These findings provide novel insights into the hormonal regulation of PIWIL1 in endometrial cancer and the PIWIL1’s role in estrogen-stimulated endometrial carcinogenesis.

Video Abstract. (MP4 41319 kb)

## Background

Endometrial carcinoma could be broadly categorized into two major types, referred to as type I and type II [1, 2]. Clinically, most endometrial cancers are type I endometrial carcinomas which are estrogen-dependent endometrioid adenocarcinomas. Estrogen exerts its biological activities by binding to estrogen receptors, mainly the ERα, which regulate the expression of a variety of genes involved in the carcinogenesis and progression of endometrial carcinoma [3–6].

PIWIL1 belongs to the PIWI family. PIWI was first identified in Drosophila as an essential factor for the self-renewal of germline stem cells [7]. Evidences showed that *PIWIL1* as an oncogene was overexpressed in several tumors including gastric cancer, lung cancer, breast cancer, hepatocellular carcinoma, soft-tissue sarcoma, adenocarcinoma of the pancreas and endometrial cancer [8–14]. Most of the studies have focused on the role of PIWIs in gonadal development [15–19]. Estrogen has been suggested to play an important role in gonadal development. Several studies found that estrogen could regulate the expression of the PIWI family [20, 21]. In our previous study, we found that the expression of PIWIL1 was higher in ERα-positive endometrial cancer cell lines and tissues [9]. These publications give clues about regulation of PIWIL1 by estrogen in endometrial cancer. However, the molecular basis underlying the association between estrogen and PIWIL1 is not fully understood and remains a challenging question.

DNA methylation changes are hallmarks of every cancer type and can be early events in tumorigenesis. DNA methylation alterations may result in gene expression changes, namely gene silencing due to DNA hypermethylation and gene activation due to DNA hypomethylation. The frequent occurrence of cancer-linked DNA hypermethylation and DNA hypomethylation is associated with carcinogenesis [22]. Previous study observed the existence of promoter CpG island hypermethylation-associated silencing of *PIWIL1* in primary seminoma and non-seminoma testicular tumors [23]. Some studies also found that promoter DNA hypomethylation of *PIWIL1* could contribute to its aberrant expression in lung adenocarcinoma [24, 25]. However, the relationship between DNA methylation status of *PIWIL1* promoter and PIWIL1 expression in endometrial cancer is unknown.

Herein, we demonstrate a novel molecular mechanism by which estrogen-ERα signaling and DNA hypomethylation co-regulate PIWIL1 expression in endometrial cancer. These findings provide novel insights into the hormonal regulation of PIWIL1 in endometrial cancer and the PIWIL1’s role in estrogen-stimulated endometrial carcinogenesis.

## Methods

### Patients and samples

The study was approved by the Human Investigation Ethics Committee of the authors’ affiliated institution. The samples of endometrial carcinoma and normal endometrial tissues were obtained after written informed consent at our institution from 2017 to 2018. Thirty formalin-fixed, paraffin-embedded tissues (15 ERα-positive endometrial carcinoma and 15 ERα-negative endometrial carcinoma) were used for immunohistochemistry, and 30 fresh frozen samples were used for RT-qPCR analysis. The stages and histological grades of these tumors were established according to the criteria of the Federation International of Gynecology and Obstetrics (FIGO) surgical staging system (2009) [26]. None of the patients underwent hormone therapy, radiotherapy or chemotherapy prior to surgery.

### Reagents and antibodies

E_2_, 5-aza-deoxycytidine (5-aza-dC, the methyltransferase inhibitor), 3-(4,5-dimethylthiazol-2-yl)-2,5-diphenyltetrazolium (MTT) were purchased from Sigma (St. Louis, MO). ICI 182,780 (ERα specific antagonist) was purchased from Tocris (Ellisville, MO). Rabbit polyclonal to anti-PIWIL1 antibody (ab105393) and Rabbit monoclonal to anti-ERα antibody (ab32063) were purchased from Abcam. GAPDH (#5174, CST) was used as an internal control.

### Immunohistochemistry, RNA extraction, RT-qPCR and western blotting

Immunohistochemistry, RNA extraction, RT-qPCR and western blotting were performed as described previously [9, 27]. Primers sequences for RT-qPCR were shown in Additional file 1: Table S1. Evaluation of PIWIL1 and ERα staining was performed according to semi quantitative immunoreactivity scores [9].

### Cell preparation and culture conditions

The human endometrial carcinoma cell lines, Ishikawa, RL95–2 (ERα-positive) and HEC-1B (ERα-negative) [28] were purchased from the Chinese Academy of Sciences Committee Type Culture Collection (Shanghai, China) and maintained in DMEM/F12 (Gibco, Auckland, NZ) supplemented with 10% fetal bovine serum (FBS) (Gibco, Carlsbad, CA). Prior to treatment with different concentrations of E_2_ or 10^− 7^ mol/L ICI 182,780, cells were cultured at the same density in serum-free medium for 72 h to minimize the influence of FBS. For demethylation studies, cells were treated with 5 μM 5-aza-dC for 72 h.

### Cell transfection

The ERα expressing plasmid (exERα) and its control vector (EV), and the shRNA against ERα (shERα) and its control vector (shNC) were all purchased from Genecreat (Shanghai). The PIWIL1 expression plasmid (exPiwil1) and its control vector (EV), and shRNA against PIWIL1 (shPiwil1) and its control vector (shNC) were all purchased from Genepharma (Shanghai). Cells were transiently transfected using Lipofectamine 2000 (Invitrogen Life Technologies; USA) according to the manufacturer’s protocol.

### Cell proliferation assay

Cells were plated in 96-well plates with 2000 cells/well and was divided into the different groups: control (EV /shNC, control vector), only in the presence of E_2_ (10^−8^mol/L), exPiwil1, shPiwil1, E_2_ plus exPiwil1 and E_2_ plus shPiwil1. Then, 20 μL MTT was added to each well before incubation at 37 °C for 4 h. Absorbance values were then measured at 490 nm using a microplate reader (Bio-Red).

### Promoter-luciferase reporter assay

A luciferase reporter assay was performed as described previously [29, 30]. *PIWIL1* promoter reporter-containing plasmid (WT: wild type, MUT: mutation of the half-ERE, DEL: deletion of the half-ERE, Fig. 3a) and ERα-expressing plasmid were purchased from ELK Biotechnology CO., LTD (Shanghai). Ishikawa, RL95–2 and HEC-1B cells were seeded into 24-well plates at a density of 1 × 10^5^ per well on the day prior to transfection. The following day, *PIWIL1* promoter reporter-containing plasmid (WT, MUT and DEL) together with internal control plasmid expressing Renilla-luciferase were co-transfected using Lipofectamine 2000 (Invitrogen Life Technologies; USA). Reporter activity was measured at 48 h post-transfection using a Dual-Luciferase Assay System (Promega; Madison, WI, USA). Besides, cells which were stimulated with or without 17β-E_2_ (10^−8^mol/L) were also co-transfected with *PIWIL1* promoter reporter-containing plasmid (WT, MUT and DEL), ERα-expressing plasmid and internal control plasmid expressing Renilla-luciferase to examine the role of ERα in PIWIL1 expression.

### Chromatin Immunoprecipitation (ChIP) assay

ChIP assays were performed using a ChIP assay kit (Millipore) as previously described in the Ishikawa cell line [27, 29, 31]. Samples were sonicated to shear DNA to an average fragment size of 200-1000 bp. Precipitated DNA was analyzed by qPCR with *PIWIL1* promoter-specific primers Primer pair1, Primer pair2 and Primer pair3 (Fig. 4a and Additional file 2: Table S2) [29]. Enrichment was calculated using the 2^-△△Ct^ formula. IgG was used as negative control.

### Bisulfite sequencing PCR

According to the manufacture’s instruction, genomic DNA was isolated using the DNA Extraction Mini Kit (TIANGEN Biotech, Beijing, China) and bisulfite modification was performed with the EZ DNA Methylation Gold Kit (ZYMO Research, Los Angeles, CA, USA). Primer sequences for bisulfite sequencing of the *PIWIL1* fragment were 5′-GGTGTTTTGGGGGGTTAGG-3′ (forward) and 5′-ACCTCCCAAAACCTCCTTC-3′ (reverse), which were used to amplify a 376 bp product. This area contains 40 CpG sites. The PCR conditions were: denaturation at 95 °C for 4 min, 35 cycles at 95 °C for 30s, 56 °C for 30s, and 72 °C for 30s. PCR products were purified directly using the TIAN gel Midi Purification Kit (TIANGEN Biotech, Beijing, China) and ligated into the pGEM-T easy vector (Promega Corporation, Madison, WI, USA). Purified plasmid DNA containing the *PIWIL1* sequence was sequenced.

### Statistical analysis

All data analyses were performed using the software package SPSS v. 18.0 (SPSS Inc., Chicago, IL, USA). Data were presented as mean ± SD and Student’s t-test was used for comparison between two groups. Correlation analysis was performed with the Spearman’s test. *p* values < 0.05 were considered statistically significant. All experiments were performed at least three times.

## Results

### PIWIL1 expression induced by E_2_ in endometrial cancer cells

**Fig. 1:**
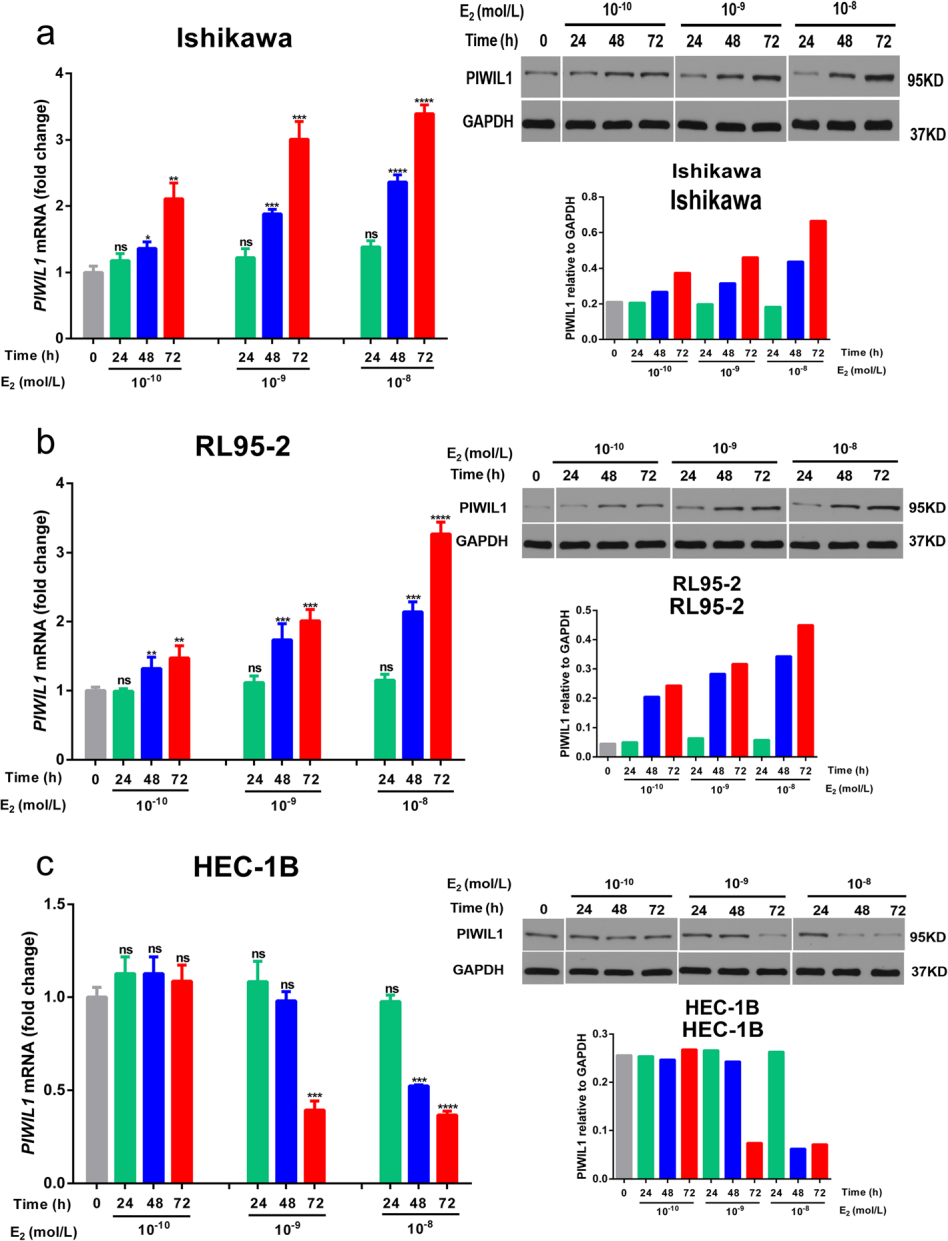
Effects of estrogen on PIWIL1 expression in endometrial cancer cells. **a,b,c** Ishikawa, RL95–2 and HEC-1B cells were treated for treated for different times(24 h, 48 h, 72 h) with different concentrations of E_2_ (10^− 10^ ~ 10^− 8^ mol/L). *PIWIL1* mRNA and protein levels were measured by RT-qPCR and western blot. Data were represented as means ± SD for three independent experiments (RT-qPCR). *p < 0.05, **p < 0.01, ***p < 0.001, ****p < 0.0001 and ns, not significant versus control group. GAPDH was used as an internal control (western blot)

We first utilized different concentrations of E_2_ (10^− 10^ ~ 10^− 8^ mol/L) to stimulate three endometrial cancer lines and analyzed the expression of PIWIL1 at different times (24 h, 48 h, 72 h). We found that the expression of PIWIL1 was up-regulated by E_2_ treatment in a time- and dose-dependent manner in Ishikawa and RL95–2 cells (Fig. 1a and b). For HEC-1B cells, we found that estrogen regulated the expression of PIWIL1 in another time- and dose-dependent manner (Fig. 1c).

### Involvement of the ERα in E_2_-induced PIWIL1 expression

**Fig. 2:**
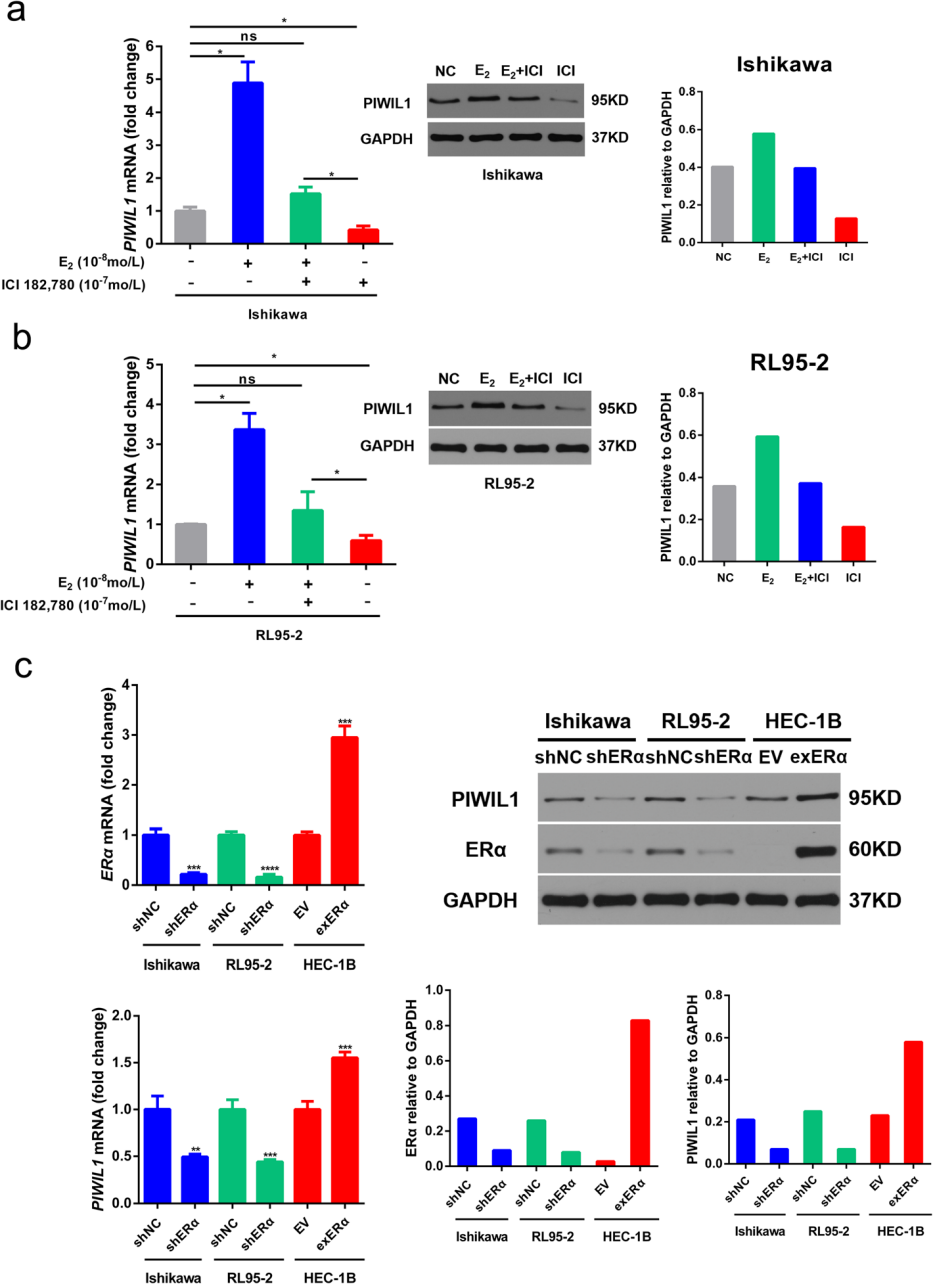
Involvement of the ERα in E_2_-induced PIWIL1 expression. **a** and **b** The ERα antagonist ICI 182,780 (10^− 7^ mol/L) was used to examine ERα in E_2_-induced PIWIL1 induction in Ishikawa and RL95–2 cells. **c** Ishikawa and RL95–2 cells were transfected with ERα shRNA (shERα) and HEC-1B was transfected with ERα expressing vector (exERα). The mRNA and protein levels of *PIWIL1* and *ERα* were then assayed using RT-qPCR and western blot. Data were represented as means ± SD for three independent experiments (RT-qPCR). *p < 0.05, **p < 0.01, ***p < 0.001, ****p < 0.0001 and ns, not significant versus control group. GAPDH was used as an internal control (western blot)

Using an ERα antagonist (ICI 182,780, 10^− 7^ mol/L), we found that the increased level of PIWIL1 induced by E_2_ (10^− 8^ mol/L) was suppressed in Ishikawa and RL95–2 cells (Fig. 2a and b).

To further understand the regulatory relationship between PIWIL1 and estrogen-ERα signaling, Ishikawa and RL95–2 cells were transfected with ERα shRNA (shERα) and HEC-1B cells was transfected with ERα-expressing vector (exERα). These results showed that silencing ERα decreased the expression of PIWIL1 and overexpression of ERα increased the expression of PIWIL1 (Fig. 2c). These results suggested that estrogen-ERα signaling can up-regulate the expression of PIWIL1 in ERα-positive endometrial cancer cells.

### Effect of the half-ERE in the *PIWIL1* promoter for ERα binding

**Fig. 3:**
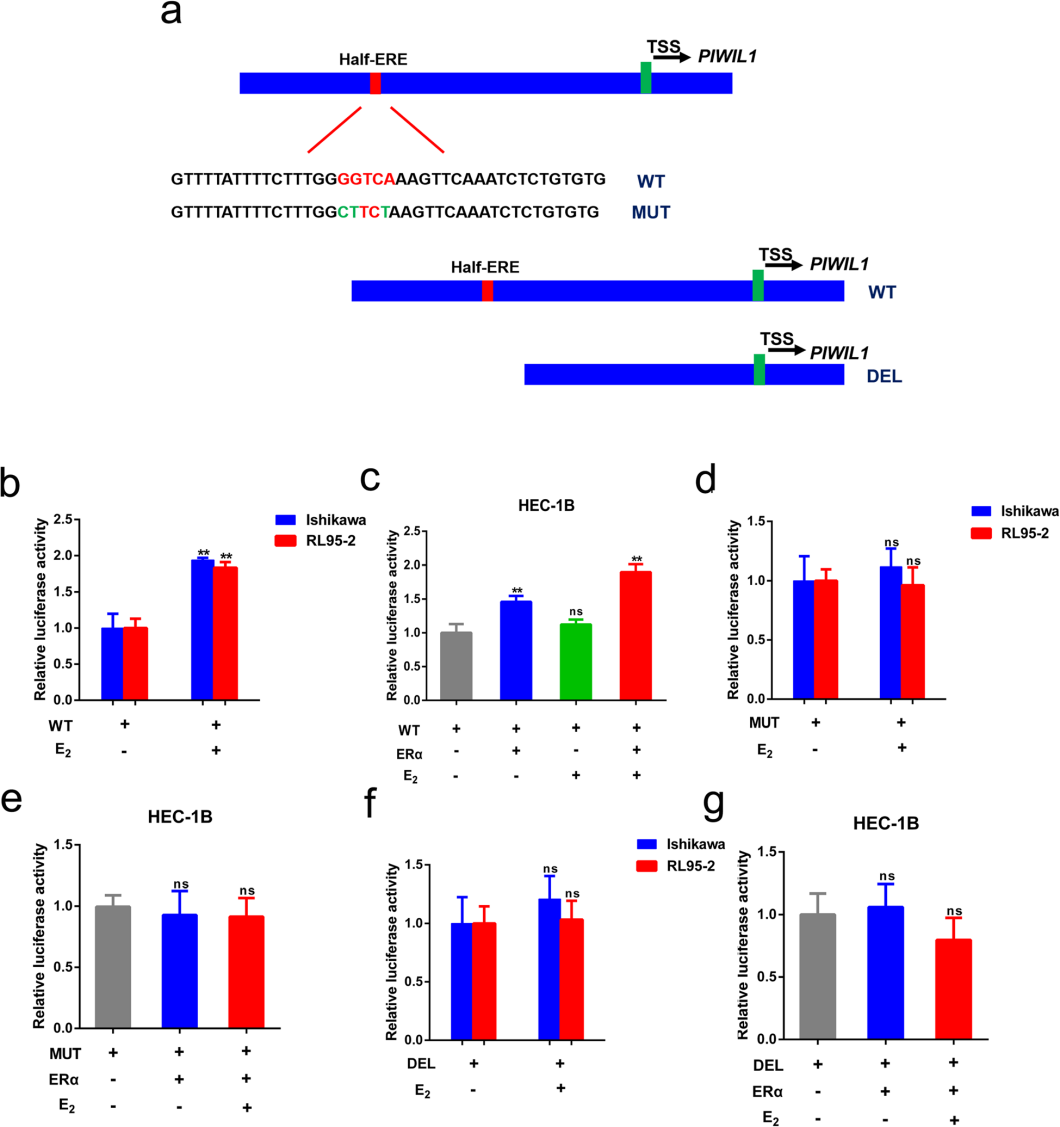
Estrogen up-regulates the transcription of PIWIL1. **a** A schematic of three *PIWIL1* promoter-luciferase reporter constructs (wild type, WT; Mutation of the half-ERE, MUT; Deletion of the half-ERE, DEL) was shown. **b** and **c** Luciferase activities of the WT reporter in endometrial cancer cell lines. **d** and **e** Luciferase activities of the WUT reporter in endometrial cancer cell lines. **f** and **g** Luciferase activities of the DEL reporter in endometrial cancer cell lines. The expression construct of ERα was co-transfected into ERα-negative cell lines. All experiments were performed at three times. **p < 0.01 and ns, not significant versus control group

Analysis of the *PIWIL1* promoter reveals a half-ERE (5′-GGTCA-3′) [32] at ~ 1112 bp upstream to its translation start site, which is surrounded by GC-rich regions. To test whether this half-ERE is involved in ERα binding onto the *PIWIL1* promoter, we constructed a *PIWIL1* promoter-luciferase reporter plasmid (wild type, WT) (Fig. 3a) and evaluated the effect of estrogen-ERα signaling on the activity of the *PIWIL1* promoter in Ishikawa, RL95–2 and HEC-1B cells. In Ishikawa and RL95–2 cells, E_2_ treatment significantly increased *PIWIL1* promoter activities as determined by luciferase assays (Fig. 3b). Without ERα expression, E_2_ did not induce any promoter activities in HEC-1B cells (Fig. 3c), indicating the necessity of ERα in the estrogen-induced *PIWIL1* promoter activity. Re-expression of ERα increased *PIWIL1* promoter activities and a combination of ERα expression vector and E_2_ treatment dramatically increased the activity (Fig. 3c). We next constructed two additional *PIWIL1* promoter-luciferase reporter plasmids (Mutation of the half-ERE, MUT [30]; Deletion of the half-ERE, DEL; Fig. 3a). We transfected MUT or DEL into Ishikawa and RL95–2 cells. E_2_ treatment didn’t increase the activities (Fig. 3d and e). After co-transfection with the ERα-expression vector and MUT or DEL into HEC-1B cells, mutation or deletion of the ERE sequence of *PIWIL1* abolished the promotion effect of estrogen-ERα signaling (Fig. 3f and g). These results further support the PIWIL1 expression is up-regulated by estrogen-ERα signaling and suggest that the half-ERE in the *PIWIL1* promoter is essential for ERα binding.

### E_2_-induced binding of the ERα onto the *PIWIL1* promoter

**Fig. 4:**
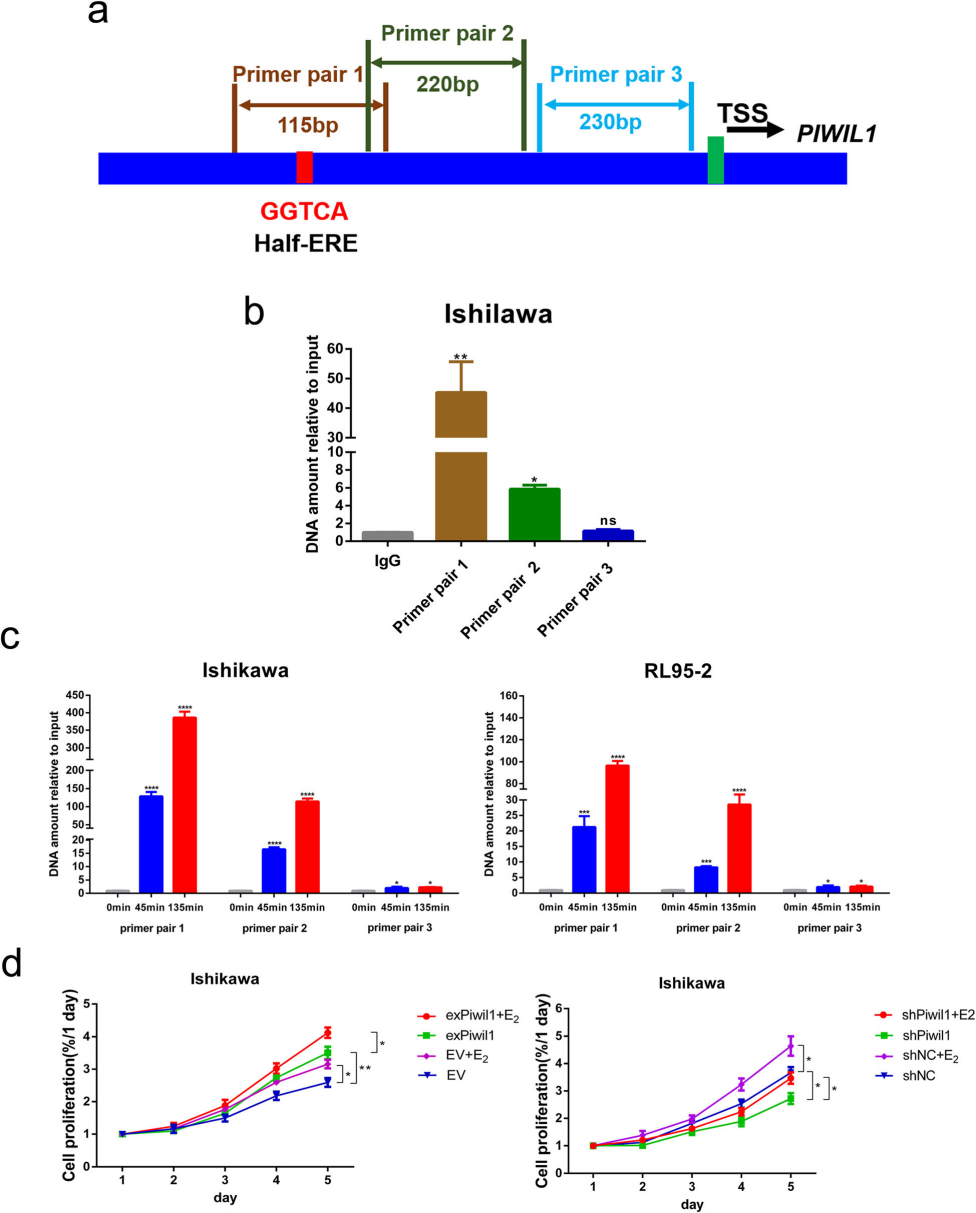
E_2_-induced binding of the ERα onto the *PIWIL1* promoter by estrogen. **a** A Schematic representation of the half-ERE in the *PIWIL1* promoter and the three pairs of primers used for ChIP-qPCR was shown. TSS: transcription start sites. **b** the *PIWIL1* promoter region was precipitated by the antibody against ERα in Ishikawa cells cultured in normal media. IgG served as the negative control. RT-qPCR was performed on ChIP samples. *p < 0.05, **p < 0.01 and ns, not significant versus control group. **c** Ishikawa cells treated with 10^− 8^ mol/L E_2_ for 0, 45, and 135 min were subjected to ChIP and the precipitated DNA was analyzed by RT-qPCR. Experimental conditions are identical to those in panel **b**, except that cells were cultured in hormone-free media for 72 h before E_2_ treatment. All experiments were performed at three times. *p < 0.05, ***p < 0.001 and ****p < 0.0001 versus control group (cells without E_2_ treatment). **d** the effects of PIWIL on E_2_-induced cell growth were determined by MTT assay. *p < 0.05 and **p < 0.01 versus control group

Then we performed ChIP-qPCR to determine whether estrogen induces the binding of the ERα onto the *PIWIL1* promoter. We designed three pairs of PCR primers used to amplify the *PIWIL1* promoter DNA: Primer pair 1, Primer pair 2 and Primer pair 3 [29]. The half-ERE was in the fragment amplified by Primer pair1 (Fig. 4a). Ishikawa cells grown in regular media were lysed to prepare the chromatin DNA and then subjected to ChIP with anti-ERα antibody. Our study showed that the DNA amplified by Primer pair 1 was the most abundant followed by Primer pair 2. These results suggest that estrogen up-regulates the transcription of *PIWIL1* by inducing the binding of the ERα onto the *PIWIL1* promoter at the half-ERE (Fig. 4b).

ERα cycles onto and off promoters of its target genes in response to E_2_ and its promoter occupancy peaks at 45 and 135 min post-E_2_ treatment [29]. We supposed that whether the binding of the ERα onto the *PIWIL1* promoter fits this model. To test this possibility, we examined the binding of the ERα at different time points after E_2_ treatment in Ishikawa and RL95–2 cells using ChIP-qPCR. ERα occupancy on the *PIWIL1* promoter was hardly detectable without E_2_ treatment but was increased by E_2_ treatment for both 45 and 135 min (Fig. 4c).

### Involvement of the PIWIL1 in E_2_-induced cell growth

To establish the role of PIWIL1 in mediating the proliferative effect of estrogen and to investigate the possible involvement of PIWIL1 in endometrial carcinogenesis, we used the MTT assay. Ishikawa cells were transfected with the PIWIL1 expression plasmid (exPiwil1), shRNA against PIWIL1 (shPiwil1) and their control vector (EV or shNC). The transfection efficiency was demonstrated in our previous study [9]. Cell growth was monitored in these cells that received no treatment or treatment with estrogen. In the cells that transfected with EV or shNC, E_2_ stimulated cell growth (Fig. 4d). In the cells that transfected with exPiwil1, cell growth was observed even with no E_2_ treatment. Treatment with estrogen further enhanced the cell growth (Fig. 4d). In the cells that transfected with shPiwil1, cell growth stimulation by estrogen was greatly attenuated (Fig. 4d). Collectively, these experiments strongly indicate that PIWIL1 is a key effector of the estrogen-induced cell growth in endometrial cancer.

### Detection of ERα and PIWIL1 in endometrial cancer tissues

**Fig. 5:**
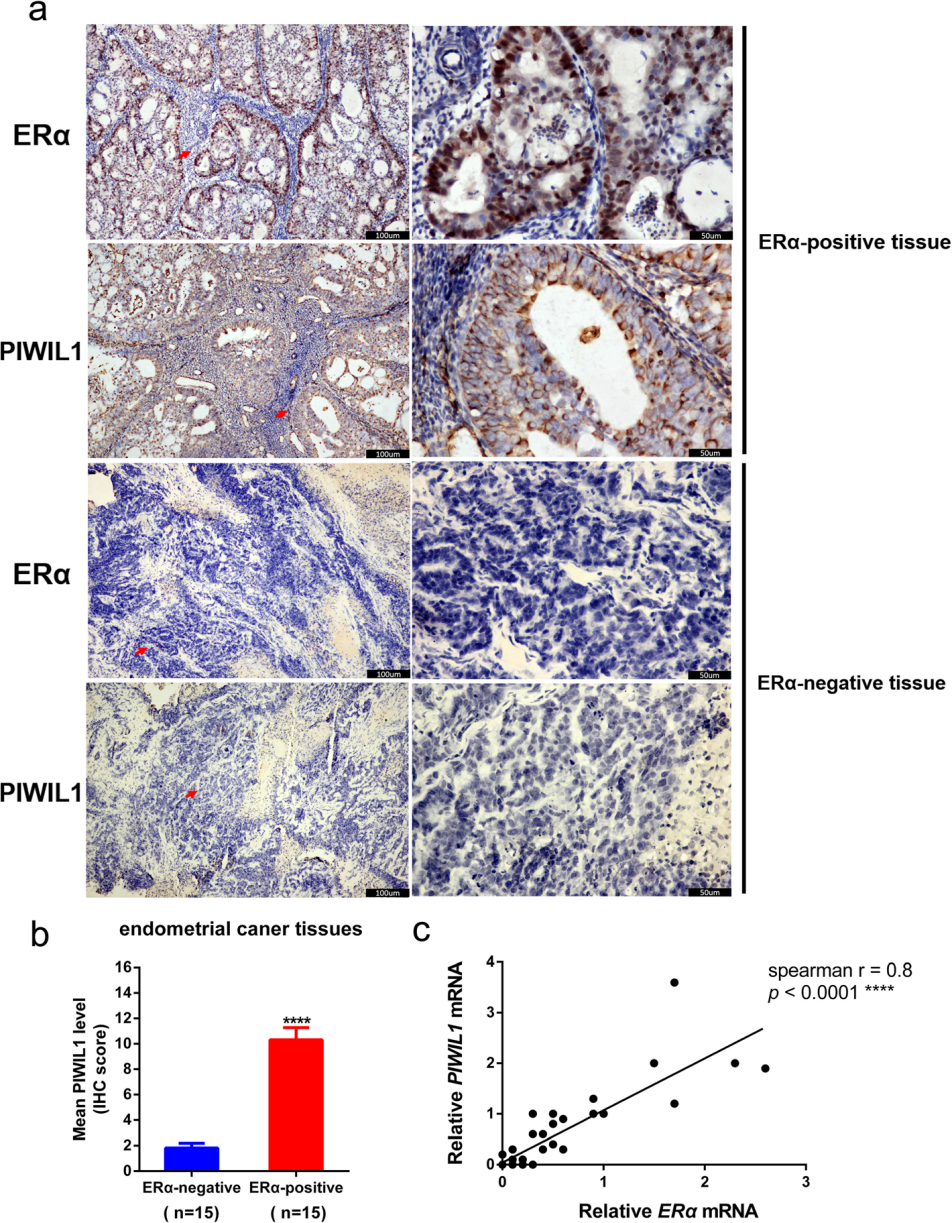
PIWIL1 and ERα expression in endometrial carcinoma tissues. **a** Example of PIWIL1 and ERα immunoreactivity in endometrial cancer tissues. Original magnification 200×, scale bar, 100 μm (left); 400×, scale bar, 50 μm (right). **b** PIWIL1 immunoreactivity scores of 15 ERα-positive endometrial cancer samples and 15 ERα-negative endometrial cancer samples. Values are the mean ± SD. ****p < 0.0001. **c** A positive correlation was detected between mRNA levels of *PIWIL1* and *ERα* in endometrial cancer samples (*r* = 0.8, ****p < 0.0001)

To further investigate whether PIWIL1 was regulated by estrogen-ERα signaling in endometrial cancer, we analyzed the expression of ERα and PIWIL1 in the 30 endometrial cancer samples (15 ERα-positive endometrial cancer samples and 15 ERα-negative endometrial cancer samples) used immunohistochemistry. In our study, PIWIL1 immunoreactivity was mainly observed in ERα-positive endometrial cancer tissues (Fig. 5a). The mean scores for PIWIL1 staining were 10.32 for ERα-positive endometrial cancer samples and 1.82 for ERα-negative endometrial cancer samples (Fig. 5b). Then the mRNA expression levels of *ERα* and *PIWIL1* were measured by RT–qPCR in 30 endometrial cancer samples. The *ERα* had a positive correlation with *PIWIL1* in endometrial cancer tissue (*r* = 0.8, ****p < 0.0001; Fig. 5c). Taken together, these results further suggest there is a relationship between PIWIL1 and estrogen-ERα signaling in endometrial cancer. We examined the RNA-seq data from the TCGA and found that there was also a positive correlation between *PIWIL1* and *ERα* expression in cervical cancer, kidney cancer, prostate adenocarcinoma, testicular germ cell tumors and cutaneous melanoma (data not shown).

### *PIWIL1* promoter hypomethylation

**Fig. 6:**
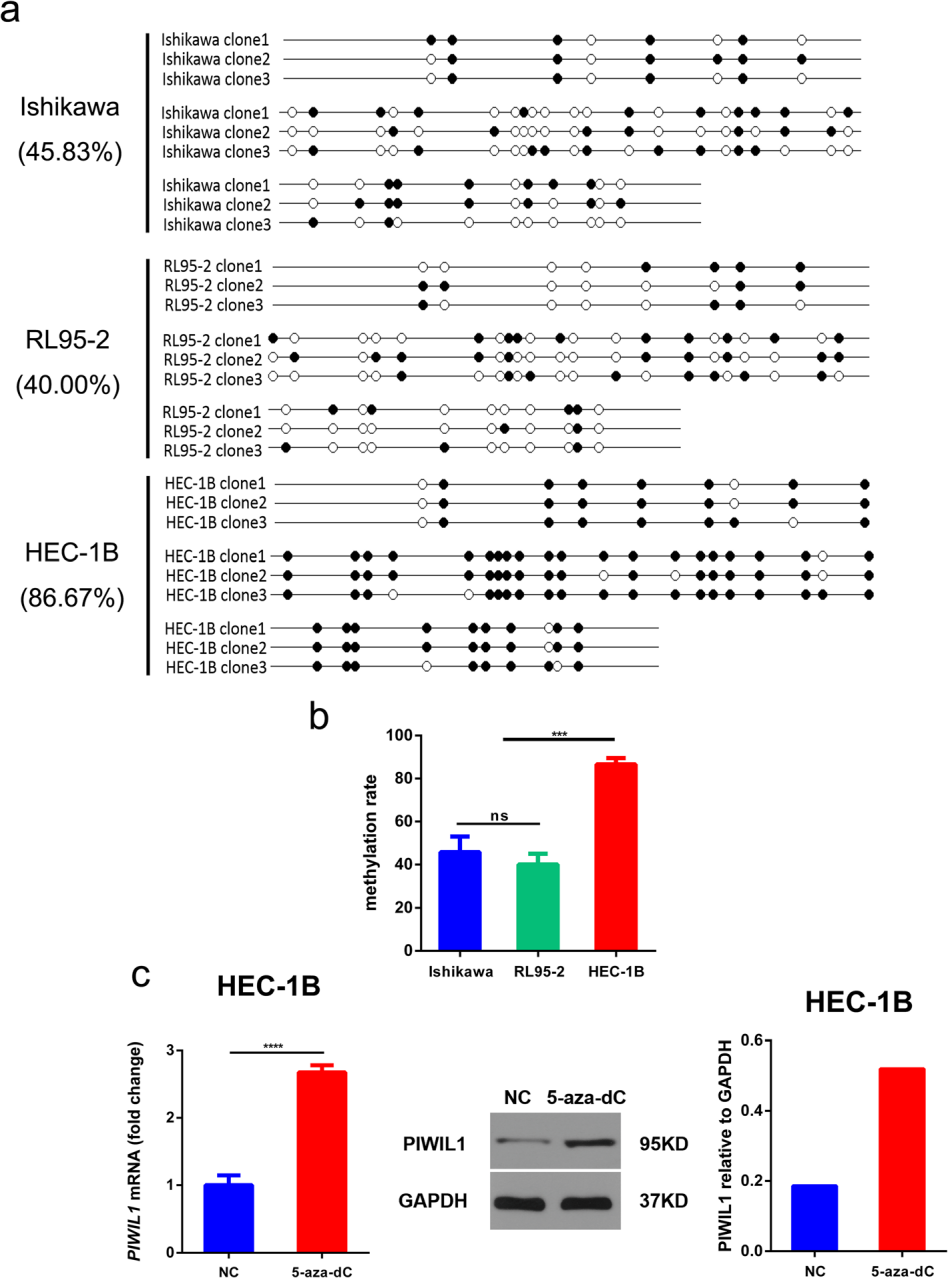
Cancer-linked hypomethylation of the *PIWIL1* promoter. **a** Results of bisulfite DNA sequencing of the *PIWIL1* upstream regulatory region in Ishikawa, RL95–2 and HEC-1B cells. Black dots symbolize methylated CpGs and white dots symbolize unmethylated CpGs. **b** The percentage of methylated CpG dinucleotides in Ishikawa, RL95–2 and HEC-1B cells. ***p < 0.001 and ns, not significant. **c** RT-qPCR and western blot showed changes in *PIWIL1* mRNA and protein expression in HEC-1B cells after treatment with 5-aza-dC. Data were represented as means ± SD for three independent experiments (RT-qPCR). ****p < 0.0001 versus control group. GAPDH was used as an internal control (western blot)

To understand why PIWIL1 is activated in endometrial cancer, we examined the methylation status of *PIWIL1* promoter in Ishikawa, RL95–2 and HCE-1B cells. We performed direct sequencing analysis of a 376-bp fragment including 40 CpG dinucleotides in the *PIWIL1* promoter at ~ 225 bp upstream to its transcription start site and at ~ 511 bp downstream to the half-ERE binding site. Differential methylation was observed in 40 CpG dinucleotides of the promoter in the three endometrial cancer cells (Fig. 6a). We found that the percentage of methylated CpG dinucleotides in Ishikawa, RL95–2 and HEC-1B was 45.83, 40.00 and 86.67%, respectively (Fig. 6b), suggesting that the reactivation of PIWIL1 expression in endometrial cancer is associated with hypomethylation of the *PIWIL1* promoter. Moreover, we assessed the expression of *PIWIL1* mRNA and protein after treatment with 5-aza-dC in HEC-1B cells. After treatment with 5-aza-dC, we found that HEC-1B cells showed reactivation of both *PIWIL1* mRNA and protein expression (Fig. 6c).

## Discussion

PIWIL1 is a member of the PIWI proteins, which are involved in stem cell self-renewal, division, spermatogenesis, RNA silencing, and translational regulation [33]. Cancer cells share several characteristics with stem cells, such as rapid proliferation and virtually infinite self-renewal. Therefore, it is not surprising that germline factors would also be involved in carcinogenesis [34]. Our previous study revealed *PIWIL1* as an oncogene in endometrial cancer [9, 35]. In the present study, for the first time, we wanted to elicit the potentially molecular mechanism involved in regulating the expression of PIWIL1 in endometrial cancer.

Several studies found that estrogen could regulate the expression of the PIWI family [20, 21]. In our previous study, we found that the expression of PIWIL1 was higher in ERα-positive cell lines (Ishikawa and RL95–2). Our continued examination in this study indicates that estrogen could up-regulate the expression of PIWIL1 in ERα-positive endometrial cancer cells (Fig. 1). However, our results also found that estrogen showed the function of down-regulation of PIWIL1 expression in ERα-negative endometrial cancer cell line. This finding raises the possibility that there may be another mechanism in ERα-negative cell lines which warrants further investigation.

Estrogen exerts its biological activities by binding ERs, ERα and ERβ, which mediate cellular responses to hormone exposure. The adult uterus is found, in general, to have very low expression of ERβ compared with ERα [36]. Moreover, it is well accepted that estrogenic effect occurs predominantly through ERα in endometrial cancer. In this study, we further confirmed that estrogen could up-regulate the expression of PIWIL1 through ERα, which was based on multiple lines of evidence, including up-regulation of PIWIL1 by overexpression of ERα expression and down-regulation of PIWIL1 by the knockdown of ERα expression or using an ERα antagonist (Fig. 2). Therefore, it is assumed that estrogen could up-regulate the expression of PIWIL1 through ERα.

Estrogen-mediated signaling pathways can be divided into genomic signaling pathways and non-genomic signaling pathways [37]. ERα regulates genes through directly binding to DNA at estrogen response elements (EREs) or through protein-protein interactions with other direct DNA binding transcription factors, such as Sp1 and Ap1 [38–40]. Genome-wide analysis of ERα binding has uncovered thousands of loci bound by ERα after E_2_ induction and the most common motif identified at these loci is the full palindromic ERE (5′-GGTCAnnnTGACC-3′) [32]. However, the majority of bound sites of ERα do not have full palindromic sequence and usually harbor only half EREs [41, 42]. Direct promoter binding usually involves an ERE with a typical consensus sequence or a half-ERE positioned next to GC-rich regions in their promoters [29]. Stender et al. performed an unbiased search for DNA motifs enriched in the identified ER binding sites and found five most enriched DNA motifs in the WT ER binding sites including the half-ERE (GGTCA) [43]. The *PIWIL1* promoter does not have classical palindromic ERE. Instead, it contains a half-ERE (GGTCA) which is surrounded by GC-rich regions in the *PIWIL1* promoter, located ~ 1112 bp upstream to the translation start site. In our study, we found that this half-ERE was essential for the binding of the ERα onto the *PIWIL1* promoter, as revealed by Luciferase assay and ChIP-qPCR (Figs. 3 and 4a-c). However, we can’t entirely exclude the possibility that activation of the PIWIL1 by estrogen requires binding of some yet unidentified ERα-associated proteins to ERα at the *PIWIL1* promoter. Therefore, further studies are required to identify the detailed mechanism involved in regulation of ERα on PIWIL1.

Endometrioid carcinoma (Type I carcinomas) is the most common type of endometrial carcinoma. This process is commonly associated with unopposed estrogen stimulation [1]. Our observation indicated that PIWIL1 had a role in E_2_-stimulated cancer cells proliferation (Fig. 4d). In our study, IHC analysis showed that the level of PIWIL1 expression was significantly correlated with that of ERα expression. Furthermore, RT-qPCR analysis and bioinformatics analysis were performed to confirm our result (Fig. 5). This correlation further suggests that *PIWIL1* may be a downstream target of ERα and may be involved in E_2_-stimulated endometrial carcinogenesis.

Aberrant DNA methylation have been shown to be an early event in carcinogenesis in many cancers, including endometrial cancer [44, 45]. Promoters with a high density of CpGs are defined as CG-rich areas and are predominantly subject to DNA methylation. The *PIWIL1* has 5′ end CpG islands surrounding the corresponding transcription start sites. Gain of 5′ end promoter CpG island methylation for the *PIWIL1* is in association with their transcriptional silencing [23]. Promoter DNA hypomethylation of *PIWIL1* could also contribute to its aberrant expression [24, 25]. In our previous study, we found that PIWIL1 was silenced in normal endometrium and reactivated in endometrial cancer [9]. In this study, we further confirm that the expression of PIWIL1 in different endometrial cancer cell lines is associated with methylation status of the *PIWIL1* promoter (Fig. 6), indicating that the reactivation of PIWIL1 in endometrial cancer may be associated with cancer-linked hypomethylation of the *PIWIL1* promoter. Previous studies suggest that ERα-targeted gene expression is epigenetically regulated by ERα cooperating with co-activators in a classical and epigenetic manner [46]. Therefore, further studies are required to identify the detailed epigenetic mechanism involved in regulation of ERα on PIWIL1.

## Conclusions

**Fig. 7:**
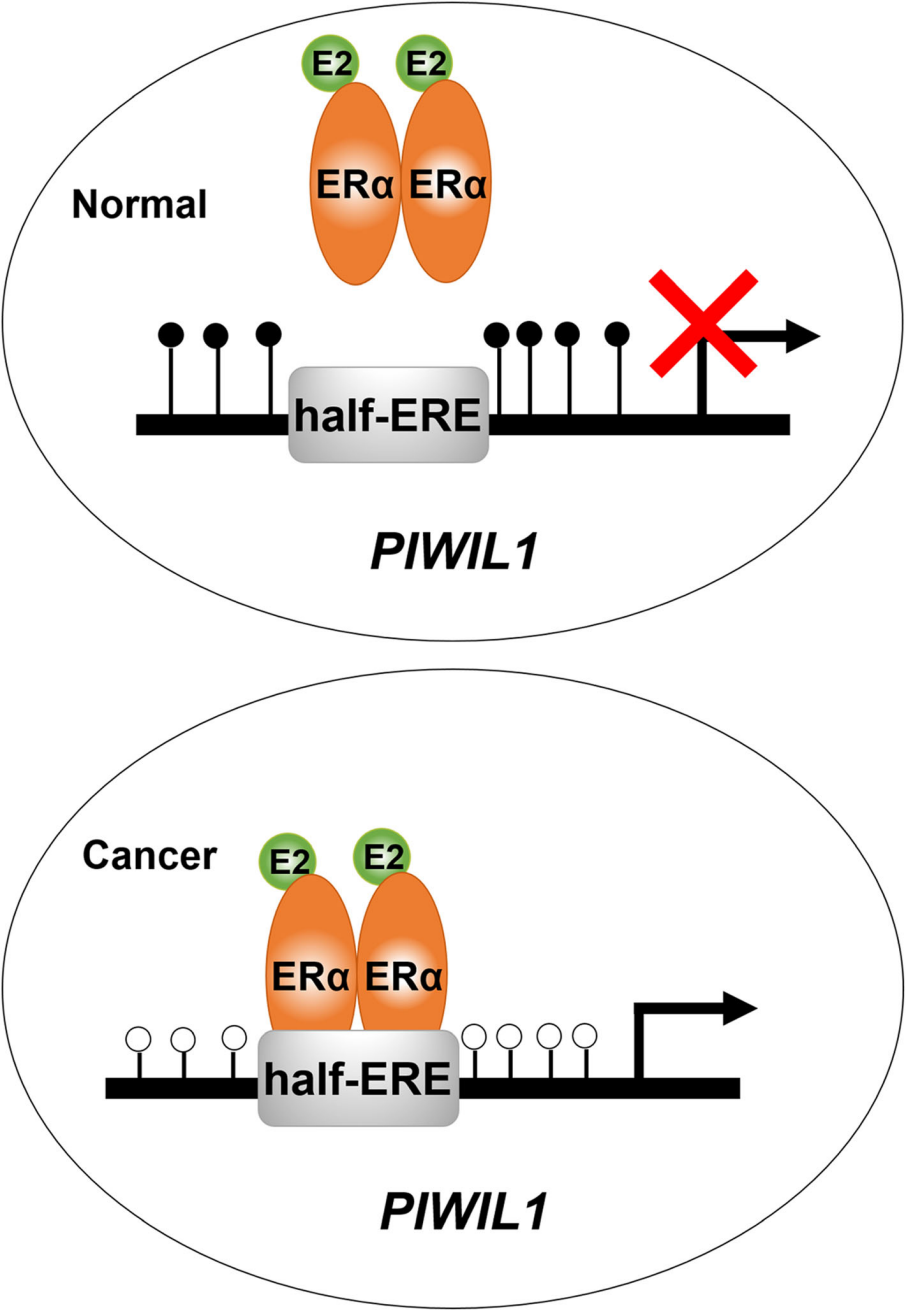
Proposed model of the cooperation between E_2_-ERα signaling and DNA hypomethylation for the regulation of stem cell protein PIWIL1. upper: In normal cells, hypermethylation of *PIWIL1* promoter prevents the binding of E_2_–ERα complex to *PIWIL1* promoter. Lower: In endometrial cancer cells, increased PIWIL1 expression regulated by E_2_–ERα signaling is due to the cancer-linked hypomethylation of the *PIWIL1* promoter

In summary, the study presented here demonstrates a novel molecular mechanism by which estrogen-ERα signaling and DNA hypomethylation co-regulate PIWIL1 expression in endometrial cancer (Fig. 7). These findings provide novel insights into the hormonal regulation in endometrial cancer and may offer novel therapeutic and preventative strategies for endometrial cancer and other hormonally-driven cancers in the future.

## Supplementary information

**Additional file 1: Table S1**. List of primers used for RT-qPCR.

**Additional file 2: Table S2**. *PIWIL1* promoter-specific primers.

## Data Availability

All data generated or analyzed during this study are included in this published article.

## Abbreviations

ER: Estrogen receptor
ChIP: Chromatin immunoprecipitation
RT-qPCR: Quantitative reverse transcription-PCR
ERE: Estrogen response element
BSP: Bisulfite sequencing PCR
FIGO: Federation International of Gynecology and Obstetrics
5-aza-dC: 5-aza-deoxycytidine
MTT: 3-(4,5-dimethylthiazol-2-yl)-2,5-diphenyltetrazolium
exERα: ERα expressing plasmid
shERα: ShRNA against ERα
exPiwil1: PIWIL1 expression plasmid
shPiwil1: ShRNA against PIWIL1
EV/shNC: Control vector
WT: Wild type
MUT: Mutation of the half-ERE
DEL: Deletion of the half-ERE
