# Supplementary material for: Estrogen-ERα signaling and DNA hypomethylation co-regulate expression of stem cell protein PIWIL1 in ERα-positive endometrial cancer cells
Source: Cell Commun Signal. 2020 Jun 5;18:84. doi: 10.1186/s12964-020-00563-4 (PMC7275358; doi:10.1186/s12964-020-00563-4)
Supplement: Supplementary file 2 — Additional file 1: Table S1. List of primers used for RT-qPCR. [file 12964_2020_563_MOESM2_ESM.doc]

Table S1. List of primers used for RT-qPCR

| *PIWIL1*-Fw | ACTAACTCCAGAGCAAAGGCAG |
| --- | --- |
| *PIWIL1*-Rev | CCTTGGTGAATCTTTTCTGTTTG |
| *GAPDH*-Fwd | CATCATCCCTGCCTCTACTGG |
| *GAPDH*-Rev | GTGGGTGTCGCTGTTGAAGTC |
| *ERα*-Fwd | TTGCTCCTAACTTGCTCTTGGAC |
| *ERα*-Rev | TCATGCGGAACCGAGATGAT |
