# Supplementary material for: Estrogen-ERα signaling and DNA hypomethylation co-regulate expression of stem cell protein PIWIL1 in ERα-positive endometrial cancer cells
Source: Cell Commun Signal. 2020 Jun 5;18:84. doi: 10.1186/s12964-020-00563-4 (PMC7275358; doi:10.1186/s12964-020-00563-4)
Supplement: Supplementary file 3 — Additional file 2: Table S2. PIWIL1 promoter-specific primers. [file 12964_2020_563_MOESM3_ESM.doc]

Table S2. *PIWIL1* promoter-specific primers.

| Primer pair1-Fw | CTGGCACCTTAGACTTGCATT |
| --- | --- |
| Primer pair1-Rev | AGTGGACTCGATCTTGGTCTG |
| Primer pair2-Fwd | ACCAAGATCGAGTCCACTGC3 |
| Primer pair2-Rev | CCTGTTCCTCTTCTGCTTCG3 |
| Primer pair3-Fwd | CGTATGGCGTACAGACACGA |
| Primer pair3-Rev | TAGTCCTGGTCCTTGCACCT |
